# Cross-Code Verification for Improved Thermophysical Properties of Argon, Krypton and Xenon Plasmas

**DOI:** 10.3390/e28070830

**Published:** 2026-07-22

**Authors:** Alberto Vagnoni, Anthony B. Murphy, Emanuele Ghedini

**Affiliations:** 1 Alma Mater Studiorum, Department of Industrial Engineering (DIN), University of Bologna, 40136 Bologna, Italy; alberto.vagnoni3@unibo.it; 2 CSIRO Technology, P.O. Box 218, Lindfield, NSW 2070, Australia

**Keywords:** plasma transport properties, plasma thermodynamic properties, plasma modeling, collision integrals, transport cross sections

## Abstract

Thermophysical properties of thermal plasmas are essential input data for computational models. The required data are usually taken from the literature without examination of their reliability. Cross-code verifications of properties are rare in the thermal plasma literature, partly due to the complexity of the calculation methods, which require a systematic treatment of large datasets, multiple computations and the adoption of different models. Here, a detailed comparison of two computational codes that use different workflows but very similar underlying methods is presented, using the example of thermophysical properties of argon, krypton, and xenon plasmas in local thermodynamic equilibrium at pressures from 1 to 100 atm. The comparison considers plasma composition, collision integrals, thermodynamic properties and, in particular, transport coefficients. The comparison allowed inconsistencies and errors to be identified and corrected, resulting in improved thermophysical properties of argon, krypton, and xenon. Furthermore, transport coefficients obtained from state-of-the-art intermolecular potentials were compared with those obtained from the simpler phenomenological potential, demonstrating good agreement, including at high pressures.

## 1. Introduction

Accurate modeling of thermal plasma flows requires the determination of thermodynamic properties and transport coefficients. These quantities enter directly into the governing equations of plasma flow simulations (i.e., mass, momentum, and energy conservation coupled with electromagnetic field equations), and their accuracy strongly influences the predictive capabilities of such numerical simulations [1,2,3,4].

These datasets are complex to compute for thermal plasmas, as they require the collection and handling of large volumes of data that are then used in computational codes employing a range of algorithms and methods. Overcoming potentially error-prone procedures, complex workflows, and the risk of numerical instabilities and inaccuracies requires a systematic and methodical approach.

To complicate matters even further, studies published in the literature often do not, for reasons of brevity and clarity, provide full details of the data used in calculations. Generally, the sources of the partition functions and collision integrals are given [5,6]. The former are required to calculate the equilibrium composition and the thermodynamic properties of plasmas, while the latter are necessary to calculate the transport coefficients. Researchers are required to trace back through the literature to identify the original data sources and, in most cases, recalculate partition functions and collision integrals from data such as atomic levels and intermolecular potentials presented in tables, figures, and/or equations. An additional problem is that data such as energies of formation and polarizabilities of chemical species are sometimes not specified. Although there are a few databases for such properties [7], the data contained are usually limited or incomplete and often differ.

To the authors’ knowledge, no direct cross-code verification effort has been proposed in the thermal plasma community for computing equilibrium composition, thermodynamic, and transport properties. Almost all studies in the literature present only final results. While some of these results are generally compared with published values, there is usually no examination of the accuracy of the data sources or the required computations. In this context, this work presents a cross-code verification between the PPFM (Plasma Properties For Many) software tool [8,9] and an independently developed plasma property code used to calculate properties for a wide range of plasma compositions [10]. Although this work is, strictly speaking, a cross-code verification study, comparison with a long-established benchmark dataset [10], which has been widely employed in the literature [11,12], provides additional indirect support for the physical credibility of the results within the domain of applicability of both implementations.

The following sections describe the workflow and methods used during cross-code verification, and the discrepancies identified, highlighting the importance of reproducibility in complex problems such as this one and the differences between the two codes.

Then, the reliability of the widely used phenomenological potential developed by Pirani et al. [13,14] is assessed, including at high pressures, by comparing transport properties calculated using collision data obtained with this potential and with traditional methods. Finally, improved transport properties for argon, krypton and xenon plasmas at pressures from 1 to 100 bar are presented.

## 2. Cross-Code Verification of High-Pressure Argon, Krypton and Xenon Plasmas

Cross-code verification aims to independently reproduce published datasets, calculations, and numerical simulations using different computational frameworks to assess their robustness and identify potential sources of discrepancy. When benchmark datasets from the literature are considered, this approach also provides validation against established reference calculations. Unlike direct comparison of final results alone, this approach allowed us to perform a detailed inspection of the entire computational chain, from partition functions to equilibrium composition and thermodynamic properties, and from interatomic potentials, and momentum-transfer cross sections to collision integrals and, finally, transport coefficients. All data for partition functions and transport cross sections, and parameters for the interaction potentials were traced back to, and where necessary, computed from, the data presented in the references provided in [10].

The pure gases modeled were argon, krypton, and xenon considering Ar, ${\mathrm{Ar}}^{+}$, ${\mathrm{Ar}}^{2+}$, ${\mathrm{Ar}}^{3+}$, ${\mathrm{Ar}}^{4+}$; Kr, ${\mathrm{Kr}}^{+}$, ${\mathrm{Kr}}^{2+}$, ${\mathrm{Kr}}^{3+}$, ${\mathrm{Kr}}^{4+}$; and Xe, ${\mathrm{Xe}}^{+}$, ${\mathrm{Xe}}^{2+}$, ${\mathrm{Xe}}^{3+}$, ${\mathrm{Xe}}^{4+}$ respectively, plus the electron $e^{-}$ in all cases. Temperatures from 300 to 30,000 K and pressures from 1 to 100 bar were considered.

### 2.1. Similarities and Differences of the Two Codes

**Figure 1:**
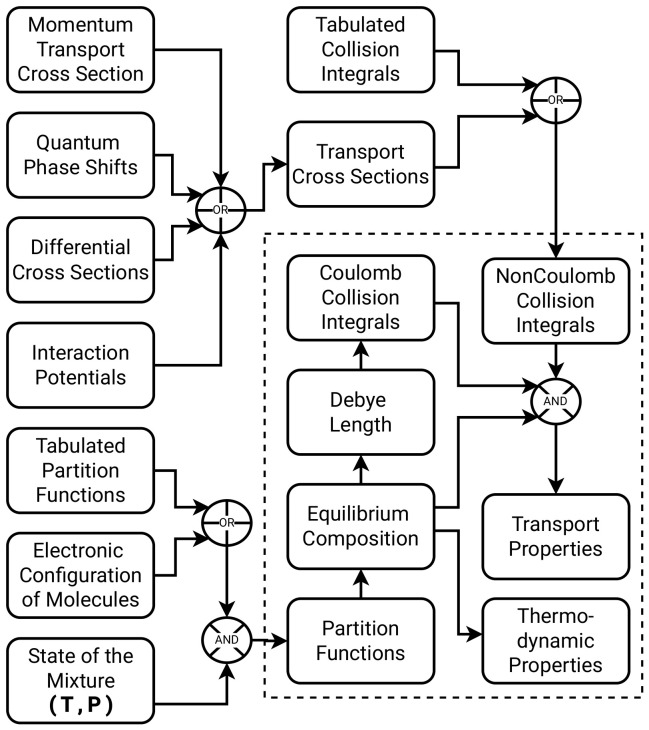
Workflow for the determination of equilibrium composition, thermodynamic properties and transport coefficients of plasmas.

Both codes used for the cross-code verification determine the equilibrium composition and the thermodynamic and transport properties of plasmas following the workflow depicted in Figure 1, which is common to the great majority of the studies reported in the literature. The code used in [10], written in Fortran 90, is organized as a collection of functions, subroutines, and databases. The code is the property of CSIRO and, while it has been shared with some collaborators, it has never been released for public use.

The Fortran 90 code divides the workflow into several modules that have to be addressed separately. The steps outside the dashed-line box in Figure 1 are performed using several separate codes, whose results are then used to populate and update the non-Coulomb collision integral and partition function databases. Using these databases, the Fortran 90 code computes the equilibrium composition, thermodynamic properties, Debye length, Coulomb collision integrals, and transport properties for a specific mixture or mixtures of input gases.

On the other hand, PPFM is implemented as a C++ object-oriented program with a structure of several classes organized in different files that encapsulate data, models, and algorithms. Furthermore, extensive use is made of C++ polymorphism to automate the workflow depicted in Figure 1 whenever possible. This makes the PPFM environment much simpler, as well as improving the readability of the source code. All data sources required for a particular problem are copied into the main file before the compilation and execution of the program. Thus, the program includes the workflow depicted in Figure 1 in its entirety, selecting the appropriate routines for the required calculations. PPFM has been released as open-source software and is available online for use [8].

The two codes implement different algorithms for calculating the equilibrium composition of the plasma. The Fortran 90 code uses the minimization of Gibbs free energy, *g*, that is

(1) $$g=\sum_{j=1}^{N}\mu_{j}N_{j}$$

where $\mu_{j}$ and $N_{j}$ are the chemical potential and the number of moles of the species *j*, respectively.

PPFM combines the Saha equations for ionization and dissociation with an algorithm based on the Mass Action Law described in [15]:

(2) $$\begin{array}{ll} \; & \frac{n_{e}n_{a^{\left(z+1\right)}}}{n_{a^{\left(z\right)}}}=2{\left(\frac{2\pi M_{e}k_{B}T_{e}}{h^{2}}\right)}^{3/2}\frac{Q_{a^{\left(z+1\right)}}^{int}\left(T_{e}\right)}{Q_{a^{\left(z\right)}}^{int}\left(T_{e}\right)}\exp\left(-\frac{\varepsilon_{a^{\left(z+1\right)}}^{f}}{k_{B}T_{e}}\right)\;, \end{array}$$

(3) $$\begin{array}{lll} \frac{n_{a}^{2}}{n_{m}}=\frac{{\left[Q_{a}^{tr}\left(T_{h}\right)\right]}^{2}}{Q_{m}^{tr}\left(T_{h}\right)}\frac{{\left[Q_{a}^{int}\left(T_{e}\right)\right]}^{2}}{Q_{m}^{int}\left(T_{h}\right)}\exp\left(-\frac{\varepsilon_{m}^{f}}{k_{B}T_{h}}\right)\;, & \; & \; \end{array}$$

(4) $$\begin{array}{cccccccccc} \sum_{i}^{species}n_{i}Z_{i}=0\;, & \; & \; & \; & \; & \; & \; & \; & \; & \; \end{array}$$

(5) $$\begin{array}{ccccccc} \;p=n_{e}k_{B}T_{e}+\sum_{i}^{heavy}n_{i}k_{B}T_{h}\;. & \; & \; & \; & \; & \; & \; \end{array}$$

where $\varepsilon^{f}$ is the energy of formation of the *m*, molecular or *a*, atomic, *z*-times ionized chemical species; $n_{i}$ and $M_{i}$ are the number density and mass of the *i*-th species, respectively; $Z_{i}$ is its charge number; and $T_{h}$ and $T_{e}$ are the heavy-species and electron temperatures in the two-temperature approach. In the present study, LTE has been considered with $T_{h}=T_{e}$. *h* and $k_{B}$ are the Planck and Boltzmann constants, respectively.

The Debye–Hückel high-pressure corrections to the calculation of the equilibrium composition are implemented in the form of corrections to the energy of formation of the chemical species to be inserted into the Saha equations in the case of PPFM, and in the form of corrections to the chemical potentials of species to which the Gibbs free energy minimization is applied in the Fortran 90 code:

(6) $$\Delta \varepsilon_{DH}^{f}=-\frac{{\left(eZ_{j}\right)}^{2}}{8\pi \epsilon_{0}\lambda_{D}}\;.$$

In addition, the lowering of the ionization potential

(7) $$\Delta I=\frac{\left(Z+1\right)e^{2}}{4\pi \epsilon_{0}\lambda_{D}}$$

is considered in calculating the internal partition function of atomic species

(8) $$Q_{j}^{int}=\sum_{i=1}^{i_{\max}}g_{i}\exp\left(\varepsilon_{i}/k_{B}T\right)$$

where $i_{\max}$ is the highest value of *i* for which $\varepsilon_{i}\leq I-\Delta I$, $\varepsilon_{i}$ is the energy of the *i*-th electronic level, *I* is the ionization potential, $\epsilon_{0}$ is the permeability of free space, and $\lambda_{D}$ is the Debye length. While both codes treat the change of the chemical potential in a similar manner, the lowering of the ionization potential is handled differently. ΔI depends on the electron density (since $\lambda_{D}$ is a function of $n_{e}$), which is determined during the calculation of the equilibrium composition. PPFM includes the lowering self-consistently within the iterations of the algorithm for the calculation of composition. In contrast, the partition functions are input data to the Fortran 90 code, and therefore cannot be modified to match the calculated electron density. Therefore, an approximate method [16] is used.

The two codes implement the same formulas for the calculation of the thermodynamic properties, their corrections, and transport properties. The interested reader can refer to [17,18,19,20,21,22,23,24,25,26] for further details. Although non-Coulomb collision integrals are calculated as input data to the Fortran 90 code, while PPFM includes this calculation in the overall calculation, essentially the same methods are used to compute the collision integrals in both codes. PPFM offers two different algorithms for the integration of deflection angles; one [27] is the same as that used for the Fortran 90 code, while the other is an adaptive alternative developed by [28], which samples the deflection angle function iteratively to avoid singularities. The two algorithms usually yield very similar results and serve as a useful diagnostic tool for this cross-code verification activity.

### 2.2. Reproducibility as a Diagnostic Tool: The PPFM Approach

The PPFM framework [8] has been specifically designed to ensure full reproducibility of plasma thermophysical property calculations. Its modular architecture explicitly separates physical models, numerical solvers, and data sources, allowing each step of the computational workflow to be independently inspected, customized and replicated in an easily reproducible workflow that minimizes the effort required of researchers.

In this study, assessment of the reproducibility of results is not treated as a verification method but as a diagnostic tool. The PPFM framework allows easy repetition of the thermophysical property calculations to assess the influence of changes in the input data sources. This feature has been crucial in reproducing published datasets, improving them where possible, and updating them to use newer literature data for properties such as ionization potentials and polarizabilities. It has also been valuable in code development. For example, the Debye–Hückel corrections were implemented within PPFM while constantly checking their correctness through comparisons with other data.

### 2.3. Identification of Inconsistencies

Using the PPFM framework, published data for argon, krypton, and xenon plasmas [10], which were calculated using the Fortran90 code, were independently replicated by identifying the data sources used to obtain the partition functions and collision integrals [17,23,29,30,31,32,33,34,35,36,37,38,39,40,41] and including the relevant partition functions, interaction potentials and cross-section data in the relevant modules of PPFM.

Differences of up to about 15% are expected in the equilibrium composition and the resulting thermodynamic and transport properties, mainly due to the different equilibrium formulations and ionization-potential-lowering models adopted by the two implementations.

In the case of argon specific heat at constant pressure, for example, percentage errors below 10% between the PPFM results and those reported in [10] were found, with exceptions around 7800 K and 16,400 K at pressures of 10 and 100 bar, for which deviations from the values given in [10] reach a maximum of 14.7% and 13.2%, respectively. In the case of argon viscosity, the PPFM values and those from the Fortran 90 code agree within 8%, with a maximum of 7.5% at 1 bar and 23,000 K; while for electrical conductivities, the discrepancy drops rapidly below 12% when ionization becomes significant around 5500 K, and settles below 3% above around 11,000 K.

Thermal conductivity deviations were higher but below about 15%, with a maximum of 15.3% at 7800 K and 1 bar. Since the thermal conductivity depends on both equilibrium composition and specific heat at constant pressure, deviations in these properties are expected to propagate to the thermal conductivity.

**Figure 2:**
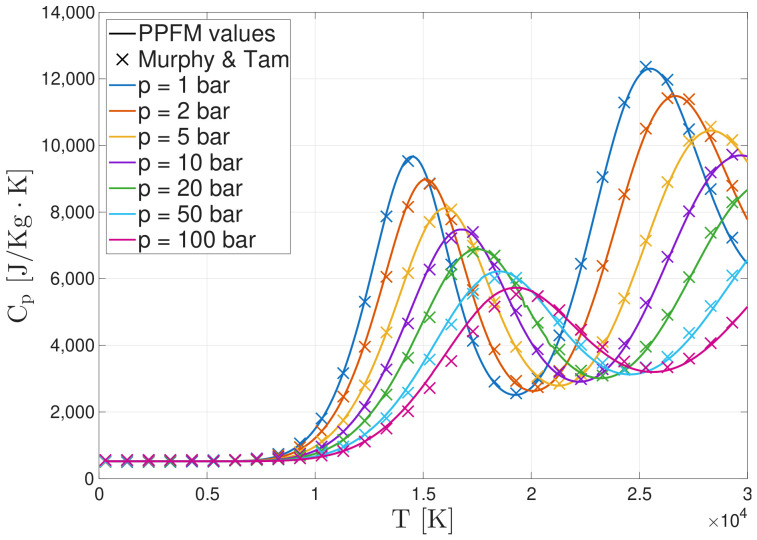
Comparison between PPFM-computed values for argon specific heat at constant pressure and data published in [10].

**Figure 3:**
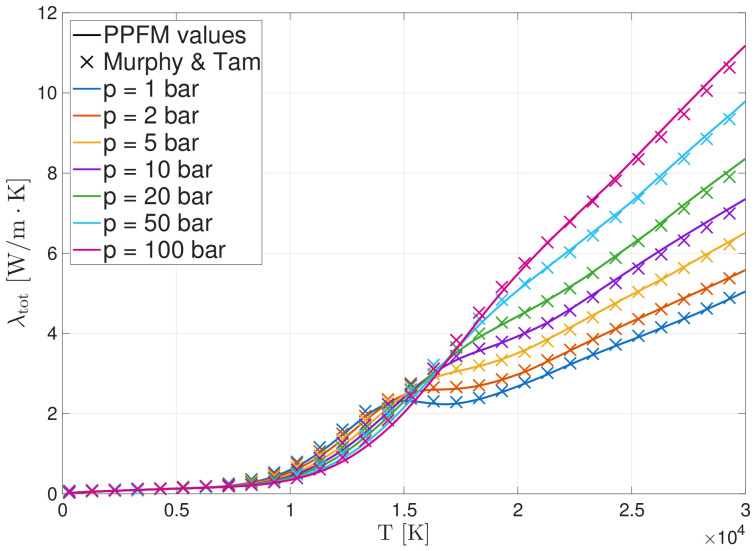
Comparison between PPFM-computed values for argon thermal conductivity and data published in [10].

**Figure 4:**
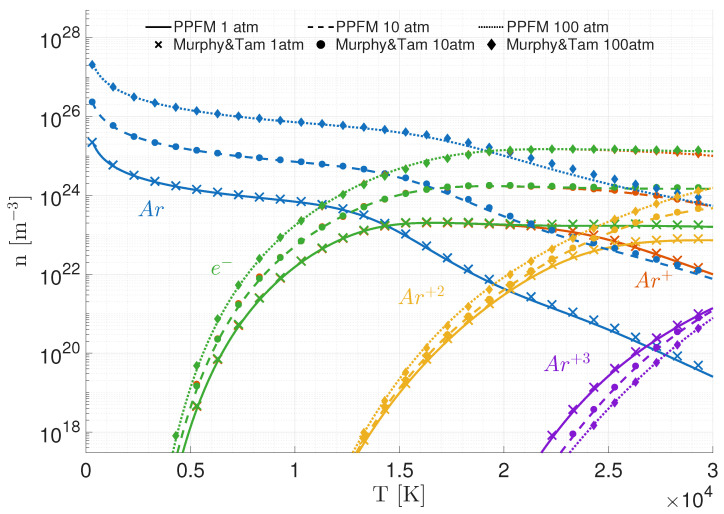
Comparison between PPFM computed values for argon equilibrium composition and data published in [10].

Equilibrium composition, specific heat at constant pressure $C_{p}$ and thermal conductivity $\lambda =\lambda_{h}+\lambda_{e}+\lambda_{r}+\lambda_{int}$, including the heavy-species and electron translational, reactive, and internal contributions, are presented for argon at different pressures in Figure 2, Figure 3 and Figure 4 for comparison.

Similar discrepancies in behavior were observed for all the remaining thermodynamic and transport properties of krypton and xenon, with the exception of krypton viscosity and xenon electrical conductivity. The former had percentage errors approximately twice those of the argon and xenon viscosities, with a smooth trend between 8000 K and 15,000 K that suggested it was a systematic error rather than a difference in models. The percentage difference in the xenon electrical conductivity dropped below 12% only above 17,700 K.

Absolute error calculations revealed maximum discrepancies of $5.89\times 10^{-5}\;\mathrm{Pa}\;s$ and 2980S/m where the computed value of krypton viscosity in [10] was $4.32\times 10^{-4}\;\mathrm{Pa}\;s$ and that of xenon electrical conductivity was 9480S/m at a pressure of 100 bar and temperatures of 13,100 K and 12,300 K, respectively.

**Figure 5:**
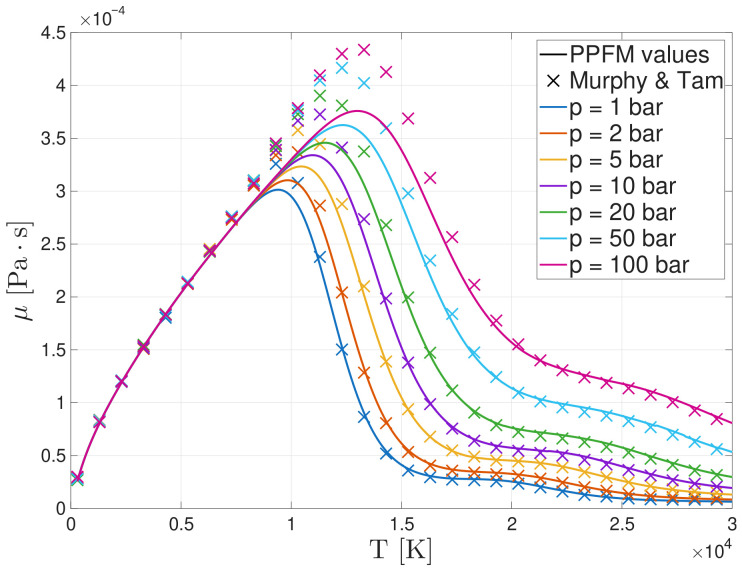
Comparison between PPFM-computed values for krypton viscosity and data published in [10].

**Figure 6:**
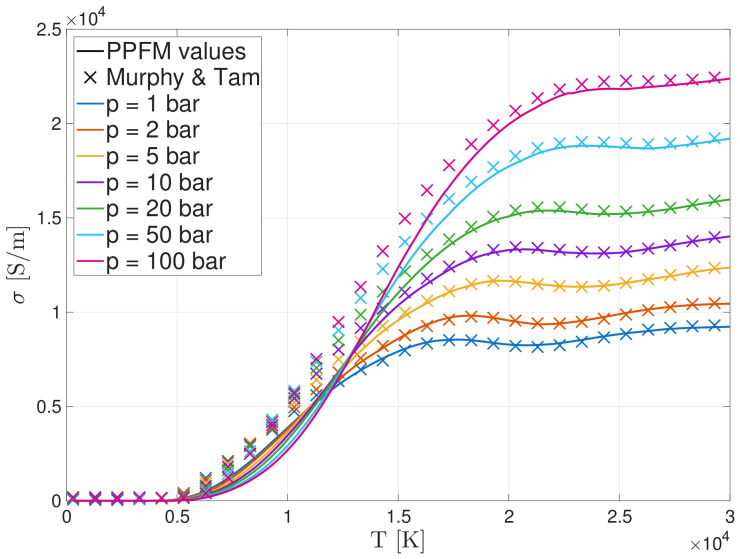
Comparison between PPFM-computed values for xenon electrical conductivity and data published in [10].

These deviations were also evident through graphical comparison between the computed values, as can be seen in Figure 5 and Figure 6, for krypton viscosity and xenon electrical conductivity, while other properties curves overlapped between PPFM-computed values and those of the Fortran 90 code, as in the argon case.

A series of tests, facilitated by PPFM’s modular structure, was undertaken to determine the source of the discrepancies. First, the transport properties were evaluated in PPFM using the phenomenological potential of Pirani et al., described in the next section. The results agreed much more closely with the PPFM values shown in Figure 5 and Figure 6 than the data from [10]. This suggested that the latter values were incorrect.

Discrepancies in the peak values of viscosity can generally be attributed to differences in the neutral–neutral collision integral at the relevant temperatures, while discrepancies in electrical conductivity at lower temperatures are due to differences in the electron–neutral collision integral. This understanding focused attention on the Kr–Kr and Xe–e^−^ collision integrals.

Taking advantage of PPFM routines that accept previously computed datasets, the collision-integral datasets from the Fortran 90 code for Kr–Kr and Xe–e^−^ interactions were incorporated into PPFM. The krypton viscosity and xenon electrical conductivity data obtained closely replicated the results presented in [10].

This indicated that discrepancies in the Kr–Kr and Xe–e^−^ collision integrals used in the two codes were indeed the source of the problem. It also confirmed that the transport coefficient calculation modules implemented in the two codes performed analogous, if not identical, calculations.

**Figure 7:**
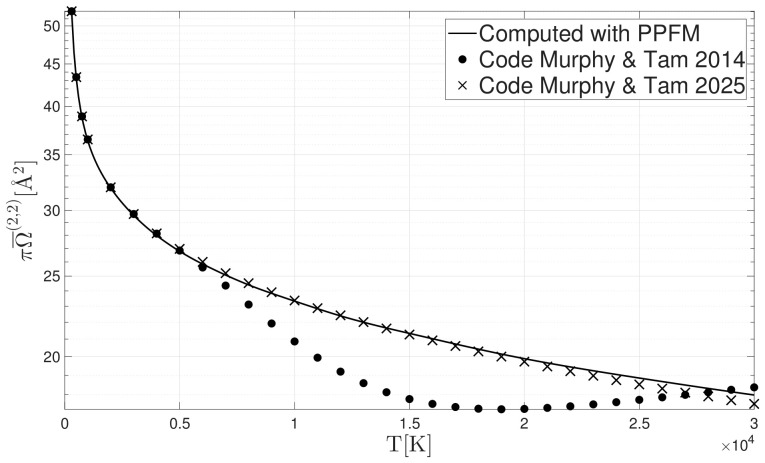
Comparison of PPFM-computed values for the collision integral of Kr–Kr interaction with those computed with the original code used in [10], before (2014), and after (2025) finding the issue through cross-code verification.

**Figure 8:**
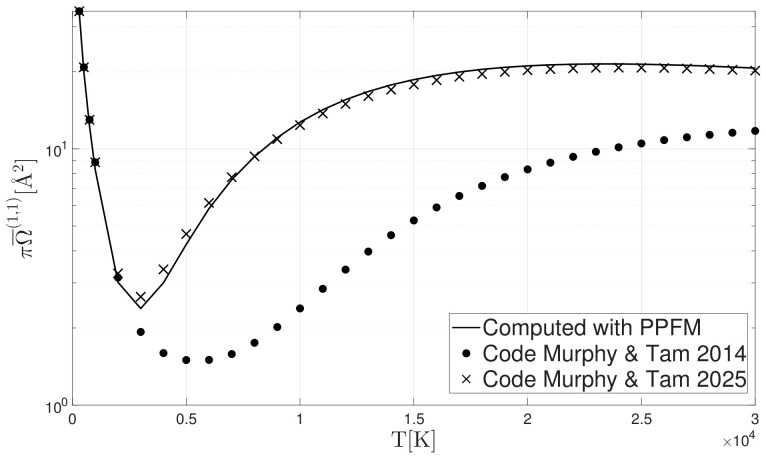
Comparison of PPFM-computed values for the collision integral of Xe–e^−^ interaction with those computed with the original code used in [10], before (2014), and after (2025) finding the issue through cross-code verification.

The differences in the collision integrals, shown in Figure 7 and Figure 8, were traced to minor typographical errors in the input files of the Fortran 90 code used for [10]. Specifically, an incorrect parameter for the energy grid of the algorithm [27] was used when integrating the Kr–Kr potential, and incorrect momentum transport cross sections (a factor of 10 too low) at energies of 2.5 eV and 5 eV were included in the input file of the code that calculated the Xe–e^−^ collision integral. Once these errors were corrected, close agreement was obtained between the collision integrals computed by the two different codes.

## 3. Updating of Datasets and Comparison of Transport Coefficients Using the Phenomenological Potential

Beyond the importance of cross-code verification in this type of study, the authors highlight that the availability of reliable reference software is essential for the accuracy of plasma thermodynamic and transport property calculations.

Such a tool should provide a consistent framework to update property datasets as new input data become available in the literature, including ionization potentials, polarizabilities, interaction potentials, cross sections, and partition functions. In this context, PPFM is designed to fulfill this role, among other functions, by enabling systematic and reproducible updates to plasma property databases.

The updated data for ionization potentials and polarizabilities of argon, krypton and xenon used for the calculations shown in the following sections are taken from [31,32], and displayed together with those used in [10], which are taken from the *Handbook of Chemistry and Physics* [30], in Table 1, Table 2, Table 3 and Table 4. The polarizabilities for the ions ${\mathrm{Ar}}^{+}$, ${\mathrm{Kr}}^{+}$, and ${\mathrm{Xe}}^{+}$ were taken from [42] and used to calculate the phenomenological potential parameters, as will be shown in the following section, and are: $\alpha_{{\mathrm{Ar}}^{+}}=0.9662\;{Å}^{3}$, $\alpha_{{\mathrm{Kr}}^{+}}=1.5974\;{Å}^{3}$ and $\alpha_{{\mathrm{Xe}}^{+}}=2.8007\;{Å}^{3}$, respectively.

As noted above, the phenomenological potential was also used to compute transport coefficients. The transport coefficients computed with collision integrals obtained by integrating this potential are compared with those obtained with traditional methods to assess the reliability of the phenomenological potential, including at high pressures.

The phenomenological potential is given by the expression

(9) $$\phi \left(r\right)=\varepsilon_{0}\left[\frac{m}{n(x)-m}{\left(\frac{1}{x}\right)}^{n(x)}-\;\frac{n(x)}{n(x)-m}{\left(\frac{1}{x}\right)}^{m}\right]$$

with parameters

(10) $$\begin{array}{ccc} x=r/R_{e} & \; & \; \end{array}$$

(11) $$\begin{array}{cc} n\left(x\right)=\beta +4x^{2} & \; \end{array}$$

(12) $$\begin{array}{cc} \; & \beta =6+\frac{5}{g_{1}\alpha_{1}^{1/3}+g_{2}\alpha_{2}^{1/3}} \end{array}$$

For neutral–neutral interactions

(13) $$\begin{array}{cc} R_{e}=1.767\frac{\alpha_{1}^{1/3}+\alpha_{2}^{1/3}}{{\left(\alpha_{1}\alpha_{2}\right)}^{0.095}} & \; \end{array}$$

(14) $$\begin{array}{cccc} \varepsilon_{0}=0.72\frac{C_{d}}{R_{e}^{6}} & \; & \; & \; \end{array}$$

(15) $$\begin{array}{cc} \; & C_{d}=15.7\frac{\alpha_{1}\alpha_{2}}{\sqrt{\alpha_{1}/N_{1}^{eff}}+\sqrt{\alpha_{2}/N_{2}^{eff}}} \end{array}$$

while for neutral–ion interactions

(16) $$\begin{array}{c} R_{e}=1.767\frac{\alpha_{i}^{1/3}+\alpha_{n}^{1/3}}{{\left(\alpha_{i}\alpha_{n}\left[1+1/\rho \right]\right)}^{0.095}} \end{array}$$

(17) $$\begin{array}{ccc} \varepsilon_{0}=5.2\frac{Z_{i}^{2}\alpha_{n}}{R_{e}^{4}}\left(1+\rho \right) & \; & \; \end{array}$$

(18) $$\begin{array}{cc} \rho =\frac{\alpha_{i}}{Z_{i}^{2}\left[1+{\left(2\alpha_{i}/\alpha_{n}\right)}^{2/3}\right]\sqrt{\alpha_{n}}} & \; \end{array}$$

where *r* [Å] is the interparticle distance, $R_{e}$ [Å] is the position of the minimum energy, β is an non-dimensional parameter depending on the hardness of interacting electronic distribution densities, $\alpha_{k}$ [${Å}^{3}$] is the electric dipole polarizability of the interacting species, $g_{i}$ are the ground-state spin multiplicities of interacting species, $\varepsilon_{0}$ [eV] is the depth of the potential well, $C_{d}$ [eV ${Å}^{6}$] is an effective long-range London coefficient, ρ is a parameter representative of the relative role of dispersion and induction attraction components in proximity to the equilibrium distance, *m* is equal to 4 for neutral–ion and 6 for neutral–neutral interactions. Finally, $N^{eff}$ is the effective number of electrons that contribute to polarization, which has been estimated using empirical formulas available in the literature [43]. For atoms and monatomic ions,

(19) $$\frac{N^{eff}}{N_{ext}}=1+\left(1-\frac{N_{ext}}{N_{int}}\right){\left(\frac{N_{int}}{N_{tot}}\right)}^{2}$$

where $N_{tot}$ is the total number of electrons, $N_{ext}$ is the number of electrons in the outermost (external) shell and $N_{int}$ is the number in the internal shells. For molecules,

(20) $$\frac{N_{eff}}{N_{tot}}=1-\frac{N_{b}\;N_{nb}}{N_{tot}^{2}}$$

where $N_{b}$ and $N_{nb}$ are the number of binding and non-binding electrons, respectively.

The parameters for the phenomenological potential are listed in Table 5. Inelastic charge transfer interactions between ions and parent atoms are also taken into account in both cases.

## 4. Results and Discussion

**Figure 9:**
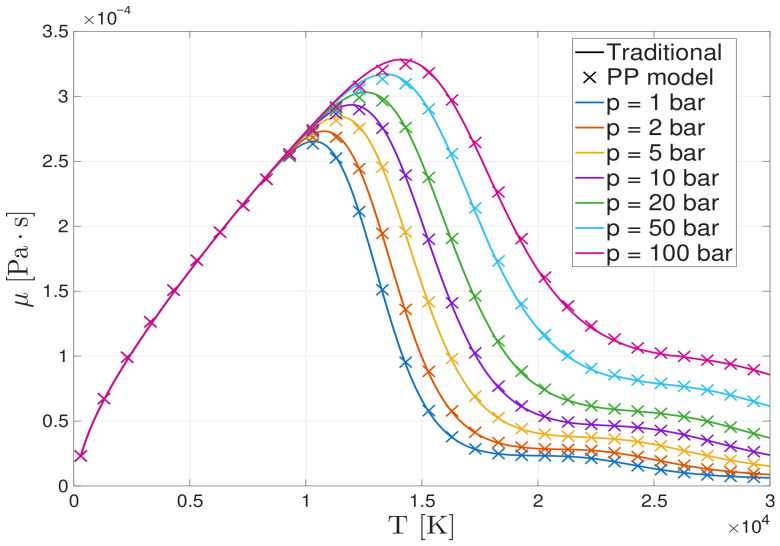
Comparison of argon viscosity at different pressures obtained with different sets of collision integrals.

**Figure 10:**
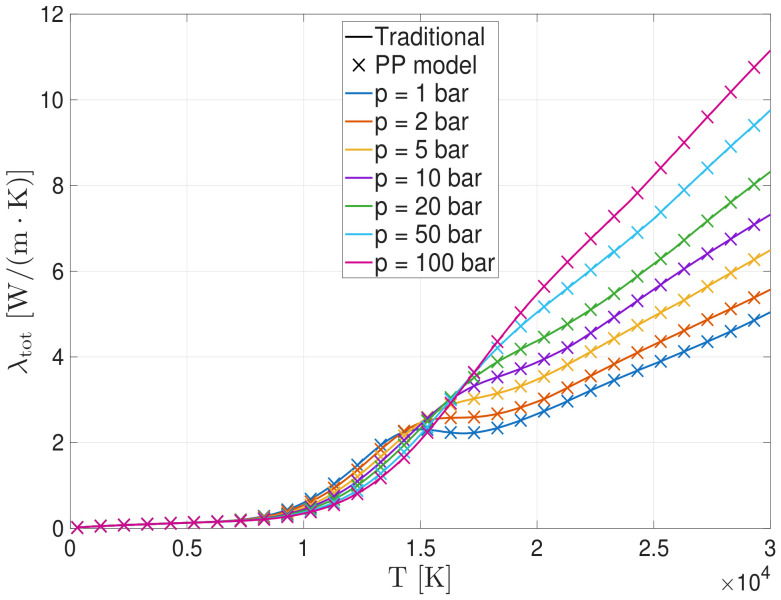
Comparison of argon thermal conductivity at different pressures obtained with different sets of collision integrals.

**Figure 11:**
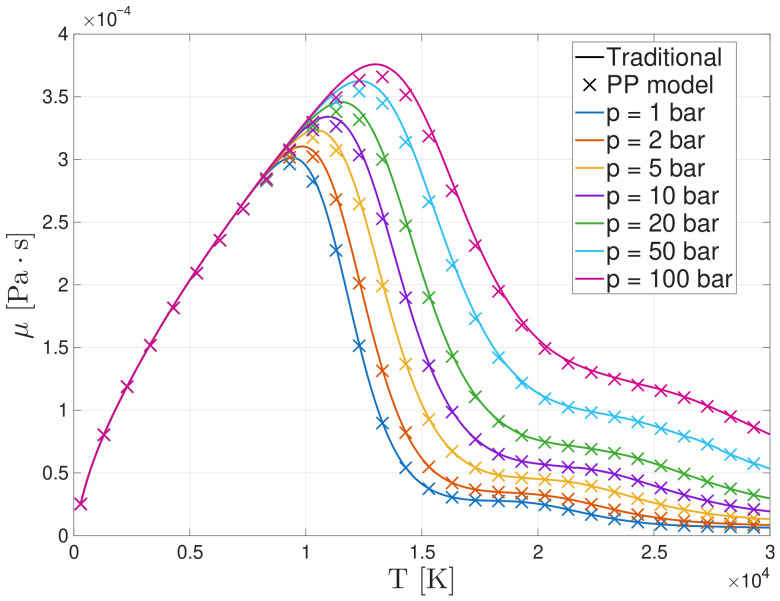
Comparison of krypton viscosity at different pressures obtained with different sets of collision integrals.

**Figure 12:**
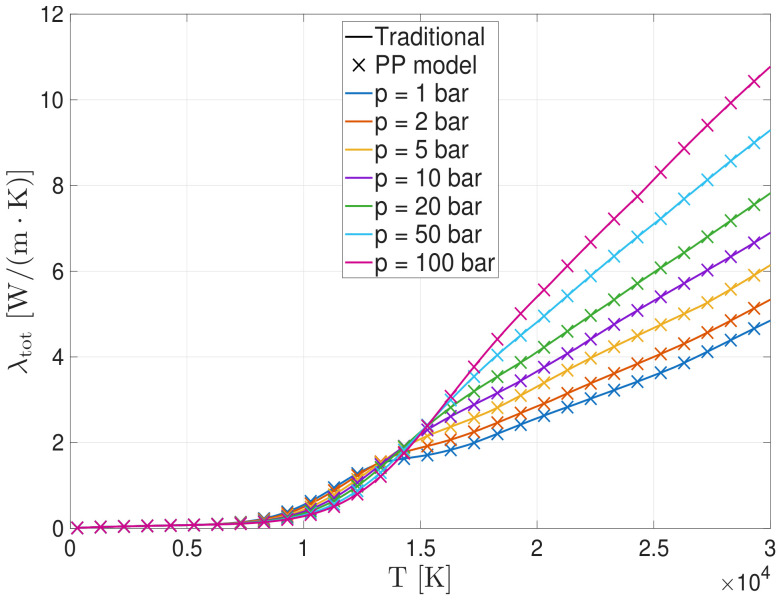
Comparison of krypton thermal conductivity at different pressures obtained with different sets of collision integrals.

**Figure 13:**
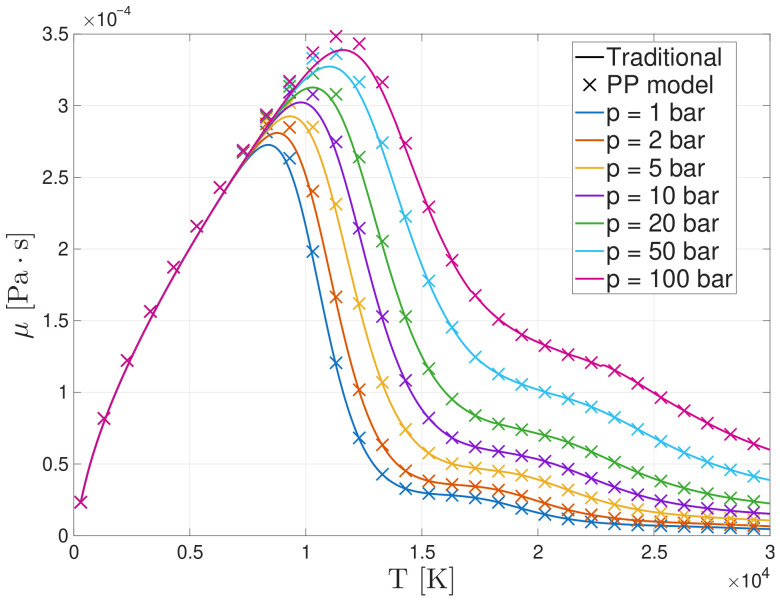
Comparison of xenon viscosity at different pressures obtained with different sets of collision integrals.

**Figure 14:**
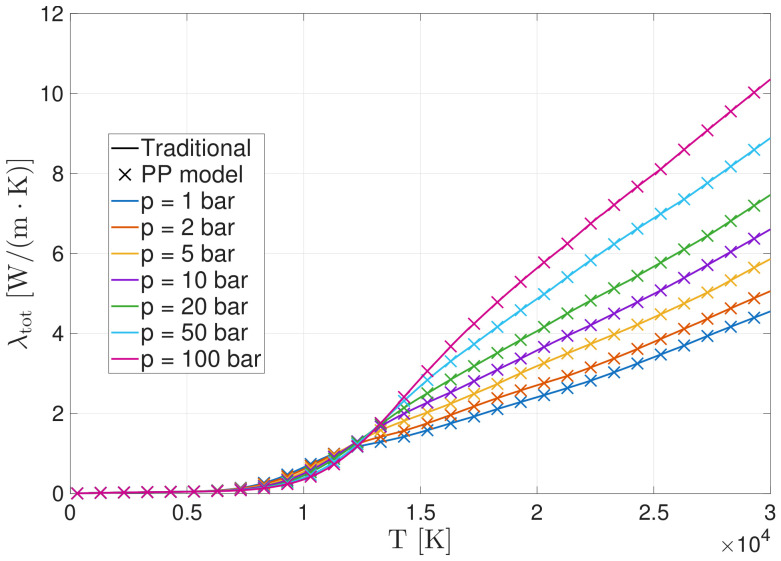
Comparison of xenon thermal conductivity at different pressures obtained with different sets of collision integrals.

The results for transport properties, with collision integrals obtained from traditional methods, of argon, krypton, and xenon at different pressures, with updated data from Table 1, Table 2, Table 3 and Table 4 are presented in Figure 9, Figure 10, Figure 11, Figure 12, Figure 13 and Figure 14, and compared with transport properties obtained with collision integrals computed integrating the phenomenological potential (PP) model.

Since the electrical conductivity depends only on electron–neutral and Coulomb cross sections, and the same sets of these cross-sections were used in [10] and in this work, no significant discrepancy was found in the electrical conductivities. Hence, a comparison of the electrical conductivities is not presented here.

The calculations of the parameters reported in Table 5, as well as both the traditional and phenomenological collision integrals, and the resulting transport coefficients, were performed separately using PPFM and the Fortran 90 code, The results were compared in the same cross-code verification fashion used in the previous sections.

As shown in Figure 9, Figure 10, Figure 11, Figure 12, Figure 13 and Figure 14, good agreement is found between the two methods for both viscosity and total thermal conductivity for all three gases, although small differences appear in the viscosity at high pressures. From Figure 9, Figure 11 and Figure 13, it can be seen that the viscosities of argon, krypton, and xenon are underestimated in the case of argon and krypton, and overestimated in the case of xenon, in the temperature range for which the gases are partially ionized, when using the phenomenological potential with respect to traditional methods.

**Figure 15:**
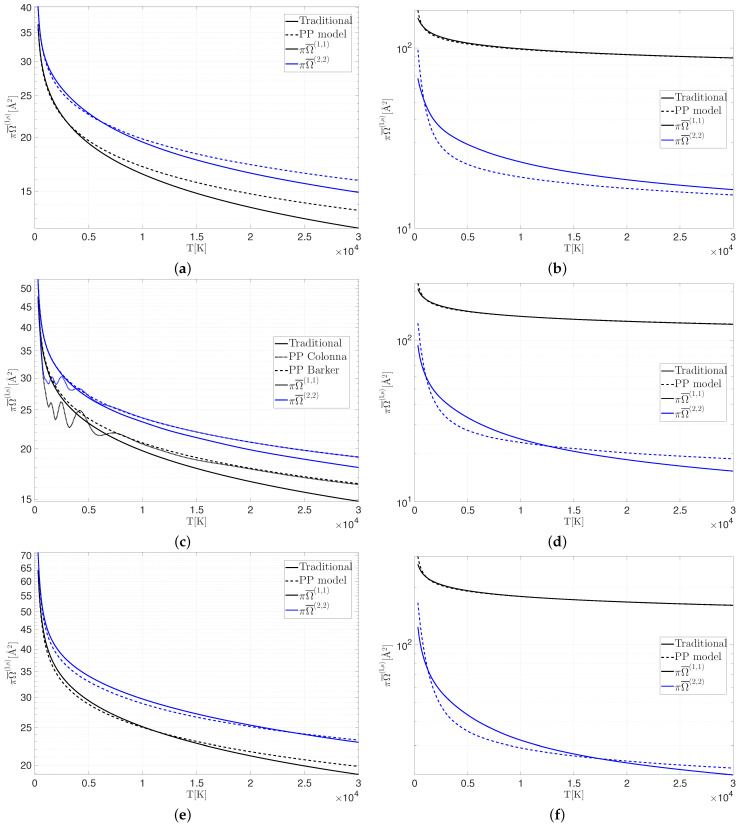
Collision integral comparisons between traditional and phenomenological potentials. (**a**) Ar–Ar. (**b**) Ar–Ar^+^. (**c**) Kr–Kr. (**d**) Kr–Kr^+^. (**e**) Xe–Xe. (**f**) Xe–Xe^+^.

The observed discrepancies are consistent with those found in the computed collision integrals, as illustrated in Figure 15. In particular, the underestimation of argon and krypton viscosities is reflected in the Ar–Ar and Kr–Kr $\Omega^{\left(1,1\right)}$ collision integrals (Figure 15a,c), for which the phenomenological potential yields higher values. Conversely, the overestimation of xenon viscosity is associated with the Xe–Xe interaction (Figure 15e), where the phenomenological potential produces lower collision integral values. This behavior is consistent since the viscosity calculated to the second-order Chapman–Enskog approximation is inversely proportional to the $\Omega^{\left(1,1\right)}$ and $\Omega^{\left(2,2\right)}$ collision integrals.

Furthermore, in the temperature range from 10,000 to 15,000 K, the dominant contribution to viscosity progressively shifts from neutral–neutral to neutral–ion interactions, with a pressure-dependent delay due to the corresponding shift in ionization of neutral species. As a result, discrepancies remain limited for argon, where overestimated and underestimated collision integrals partially compensate each other (Figure 15a,b), whereas for the examined temperature range, the Xe–Xe and Xe–Xe^+^ interactions are underestimated (Figure 15e,f), resulting in higher viscosity values around 10,000 K, as expected.

It is also worth noting the oscillatory behavior observed at low temperatures in the Kr–Kr interaction (Figure 15c) when integrating the phenomenological potential with the algorithm proposed by Colonna et al. in [28], which fails to converge at low energies, while the method of Barker et al. [27] remains monotonic. If the algorithm of [28] were used for Kr–Kr, the viscosity and heavy-species thermal conductivity values of krypton would differ by 10% to 25% in the low temperature range (600–2000 K), while, with the adoption of [27] for Kr–Kr, the differences in the computed coefficients remain below 4%, for each gas, at every pressure, for the two collision integral sets. Hence, for the data computed and shown in Figure 5 and Figure 12, the algorithm of Barker [27] has been preferred.

This again highlights the sensitivity of transport coefficient calculations not only to the interaction potentials used but also to the numerical algorithm chosen for their integration, emphasizing the relevance of the cross-code verification approaches presented here. Such verification is facilitated when open-access codes like PPFM include multiple algorithms for comparing results. Note that, since the collision integrals calculated for other interactions were unaffected by the choice of integration algorithm, [28] has been used by PPFM for computing the other transport coefficients used in this study.

## 5. Conclusions

This work demonstrates that cross-code verification is not only a powerful approach, but a necessary step to ensure the reliability of both computed datasets and research software, in accordance with standard verification and validation (V&V) practices. Achieving this level of reliability requires both close collaboration between independent research groups and transparent access to source codes and datasets, the latter being one of the guiding principles behind the development of PPFM. Furthermore, the findings of the present work indicate that the plasma modeling community would benefit from using the same cross-code verification approach for more complex molecular plasma mixtures, whose properties both PPFM and the Fortran 90 code are able to calculate [9,17].

In this context, modular platforms such as PPFM can significantly contribute to maintaining and updating plasma thermophysical property databases as new input data become available, while also supporting the construction of reference datasets for thermal plasmas to be shared across the scientific and industrial communities.

Through the reproducibility and diagnostic capabilities of PPFM, previously unnoticed inconsistencies in collision data for high-pressure noble gas plasmas were identified and corrected. The resulting improved transport property datasets for argon, krypton, and xenon plasmas provide a more reliable basis for plasma modeling applications and are available from the authors upon request.

Furthermore, the comparison between two different methods for evaluating collision integrals confirms that the phenomenological potential approach can give reliable results with reduced effort, including at higher pressures.

## Figures and Tables

**Table 1:** Ionization potentials of argon [eV].

	HCP [30]	NIST-ASD [31]
Ar	15.7596	15.7596
${\mathrm{Ar}}^{+}$	27.6297	27.6297
${\mathrm{Ar}}^{2+}$	40.74	40.735
${\mathrm{Ar}}^{3+}$	59.81	59.58
${\mathrm{Ar}}^{4+}$	65.0251	74.84

**Table 2:** Ionization potentials of krypton [eV].

	HCP [30]	NIST-ASD [31]
Kr	13.9996	13.9996
${\mathrm{Kr}}^{+}$	24.3598	24.3598
${\mathrm{Kr}}^{2+}$	36.95	35.838
${\mathrm{Kr}}^{3+}$	52.5	50.85
${\mathrm{Kr}}^{4+}$	64.7	64.69

**Table 3:** Ionization potentials of xenon [eV].

	HCP [30]	NIST-ASD [31]
Xe	12.1298	12.1298
${\mathrm{Xe}}^{+}$	21.2098	20.975
${\mathrm{Xe}}^{2+}$	32.1230	31.05
${\mathrm{Xe}}^{3+}$	–	42.20
${\mathrm{Xe}}^{4+}$	–	54.10

**Table 4:** Static dipole polarizabilities [${Å}^{3}$].

	HCP [30]	α Database [32]
Ar	1.62	1.6423
Kr	2.46	2.4865
Xe	3.99	4.0484

**Table 5:** Parameters of the phenomenological potential.

System	β	$D_{0}$ [eV]	$R_{e}$ [Å]
Ar–Ar	8.1189	0.0116	3.7945
Ar–Ar^+^	7.5838	0.1064	3.2300
Kr–Kr	7.8453	0.0176	4.0269
Kr–Kr^+^	7.3540	0.1251	3.5074
Xe–Xe	7.5686	0.0254	4.3183
Xe–Xe^+^	7.1330	0.1559	3.8466

## Data Availability

The original contributions presented in this study are included in the article/Supplementary Materials. Further inquiries can be directed to the corresponding authors.

## Supplementary Materials

The following supporting information can be downloaded at: https://www.mdpi.com/article/10.3390/e28070830/s1, Equilibrium compositions, thermodynamic properties, collision integrals and transport coefficients for argon, krypton and xenon at different pressures.
