# Supplementary figures and images for: Cross-Code Verification for Improved Thermophysical Properties of Argon, Krypton and Xenon Plasmas
Source: Entropy (Basel). 2026 Jul 22;28(7):830. doi: 10.3390/e28070830 (PMC13409251; doi:10.3390/e28070830)

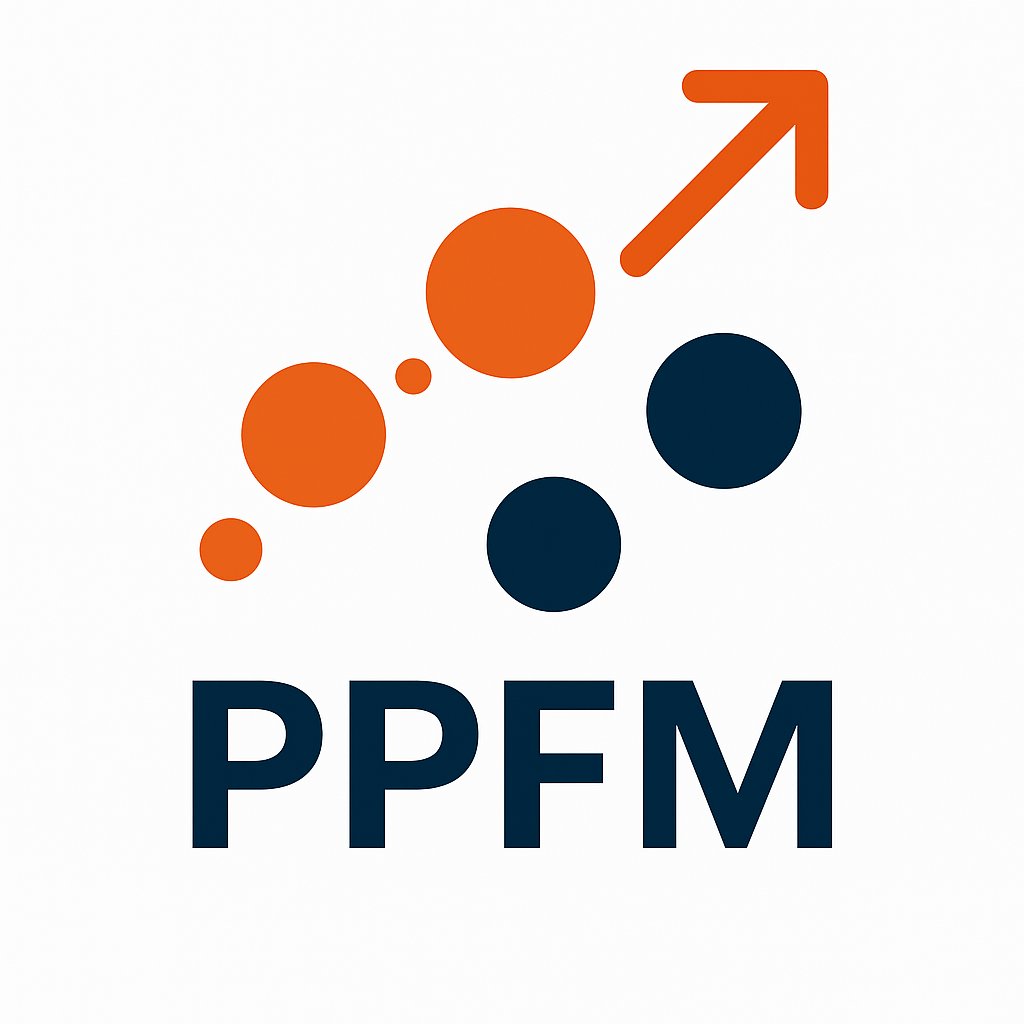

Supplement: Supplementary file 1 [file entropy-28-00830-s001.zip › Ar_Kr_Xe_Data/logo.png]
